# A Dynamic Calibration Method of Installation Misalignment Angles between Two Inertial Navigation Systems

**DOI:** 10.3390/s18092947

**Published:** 2018-09-04

**Authors:** Ming Hua, Kui Li, Yanhong Lv, Qi Wu

**Affiliations:** School of Instrumentation Science and Opto-Electronics Engineering, Beihang University, Beijing 100191, China; huaming@buaa.edu.cn (M.H.); yanhonglv@buaa.edu.cn (Y.L.); wuqi12@buaa.edu.cn (Q.W.)

**Keywords:** output information fusion, misalignment angles, dynamic calibration, inertial navigation system (INS)

## Abstract

Generally, in order to ensure the reliability of Navigation system, vehicles are usually equipped with two or more sets of inertial navigation systems (INSs). Fusion of navigation measurement information from different sets of INSs can improve the accuracy of autonomous navigation effectively. However, due to the existence of misalignment angles, the coordinate axes of different systems are usually not in coincidence with each other absolutely, which would lead to serious problems when integrating the attitudes information. Therefore, it is necessary to precisely calibrate and compensate the misalignment angles between different systems. In this paper, a dynamic calibration method of misalignment angles between two systems was proposed. This method uses the speed and attitude information of two sets of INSs during the movement of the vehicle as measurements to dynamically calibrate the misalignment angles of two systems without additional information sources or other external measuring equipment, such as turntable. A mathematical model of misalignment angles between two INSs was established. The simulation experiment and the INSs vehicle experiments were conducted to verify the effectiveness of the method. The results show that the calibration accuracy of misalignment angles between the two sets of systems can reach to 1″ while using the proposed method.

## 1. Introduction

The inertial navigation system (INS) uses inertial elements to measure the acceleration and angular velocity of carriers, so it has unique advantages of independence, information completeness, and covertness [1,2,3]. Therefore, whether in vehicles, submarines, or aircraft carriers, the INS is always one of the most important navigation systems [4,5]. As the reliability of the single INS cannot be guaranteed, vehicles are usually equipped with two sets of INSs. The navigation accuracy of the systems can be increased through the fusion of output information [6,7,8]. However, different sets of navigation systems’ direction of body coordinate cannot be completely coincident, so the output attitude information of systems will have great difference [9,10,11]. Moreover, the information fusion will be influenced seriously. Therefore, calibration of misalignment angles between systems precisely is necessary when the fusion attitude information is used [12,13].

For the calibration of misalignment angles, most of the current researches are aiming at the interior of the system, and some researches have been done on the direction of calibration misalignment angles between different sets of systems [14,15,16]. In [17], a method of calibrating misalignment angles between systems is proposed, which has high accuracy. But, this method can only calibrate the misalignment angles of horizontal axes. In [18], a method is proposed to calibrate three misalignment angles between the horizontal and vertical axes of two INSs, but this method needs the help of the three-axis turntable, so it cannot be applied to dynamic calibration.

In this study, a new dynamic calibration method is proposed for misalignment angles between two sets of systems which can calibrate the whole misalignment angles of three axes. This method uses the speed and attitude difference between two INSs as view measurements to dynamically calibrate misalignment angles for two systems without additional information sources or other external measuring equipment such as turntable. The results show that this method can calibrate the misalignment angles between systems within 2″ and it contributes to the accurate delivery of attitude reference.

The rest of the paper is organized as follows: Section 2 defined the misalignment angles and described the carrier coordinate systems’ relationship of two INSs. In Section 3, the inertial device biases model and the dynamic calibration model was proposed and a Kalman filter was built to estimate misalignment angles between systems. Section 4 analyzed the observability of state variables from both qualitative and quantitative points. In Section 5, a simulation was conducted to verify the effectiveness of the method. In Section 6, an INSs experiment was conducted to further verify the effectiveness of the method. Finally, conclusions were drawn in Section 7.

## 2. Definition of Misalignment Angles

**Figure 1:**
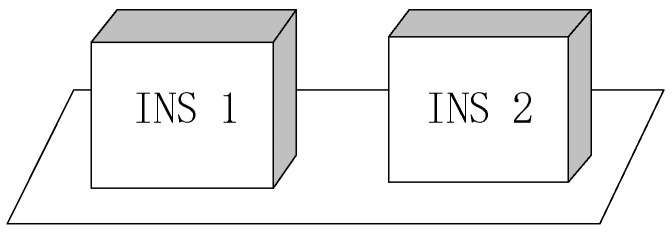
Schematic diagram of system installation.

Given that two INSs are mounted on the vehicle at different locations, as Figure 1 shows.

In order to present misalignment angles clearly, some coordinate systems need to be defined.

Body-frame (b-frame), which was defined as right-forward-upward directions along the carriers’ body, in this paper, $O-x_{b1},y_{b1},z_{b1}$ is the No.1 INS’s body-frame, $O-x_{b2},y_{b2},z_{b2}$ is the No. 2 INS’s body-frame.

Navigation-frame (n-frame) which was selected as east-north-upward coordinate system, the origin of which is the center of accelerometer unit and it usually expressed as $O-x_{n},y_{n},z_{n}$.

Calculating navigation-frame ($n^{\prime }$-frame) that has a small angle with navigation-frame. It usually expressed as $O-x_{n^{\prime }},y_{n^{\prime }},z_{n^{\prime }}$.

**Figure 2:**
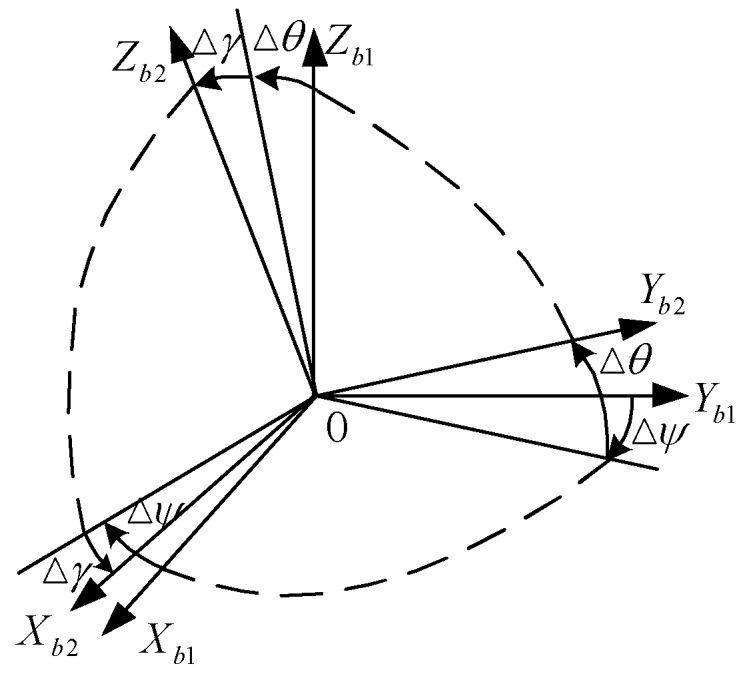
The misalignment angles between two systems.

The body-frame of the two sets of systems are not coincided because of the existence of misalignment angles between two systems, as shown in Figure 2.

The three angles Δψ, Δθ, and Δγ are misalignment angles between the two systems’ body frame. The $O-x_{b1},y_{b1},z_{b1}$ frame and $O-x_{b2},y_{b2},z_{b2}$ frame could be coincident by the transformation matrix $C_{b1}^{b2}$ and it could be expressed by three Euler angles (Δψ, Δθ, and Δγ), as follows shows:

(1) $$C_{b1\;}^{b2}=R_{2}(\Delta \gamma )R_{1}(\Delta \theta )R_{3}(\Delta \psi )$$

(2) $$R_{1}(\Delta \theta )=\left[\begin{array}{ccc} \cos(\Delta \theta )\; & 0 & \sin(\Delta \theta )\\ 0 & 1 & 0\\ -\sin(\Delta \theta ) & 0 & \cos(\Delta \theta ) \end{array}\right]$$

(3) $$R_{2}(\Delta \gamma )=\left[\begin{array}{ccc} 1 & 0 & 0\\ 0 & \cos(\Delta \gamma )\; & -\sin(\Delta \gamma )\\ 0 & \sin(\Delta \gamma ) & \cos(\Delta \gamma ) \end{array}\right]$$

(4) $$R_{3}(\Delta \psi )=\left[\begin{array}{ccc} \cos(\Delta \psi )\; & -\sin(\Delta \psi ) & 0\\ \sin(\Delta \psi ) & \cos(\Delta \psi ) & 0\\ 0 & 0 & 1 \end{array}\right]$$

After the small angle linearization, the Equation (1) can be reduced to:

(5) $$C_{b1\;}^{b2}=\left[\begin{array}{ccc} 1 & \Delta \psi & -\Delta \gamma \\ -\Delta \psi & 1 & \Delta \theta \\ \Delta \gamma & -\Delta \theta & 1 \end{array}\right]$$

## 3. Dynamic Calibration Scheme Overall Design

### 3.1. Dynamic Calibration Scheme Overview

**Figure 3:**
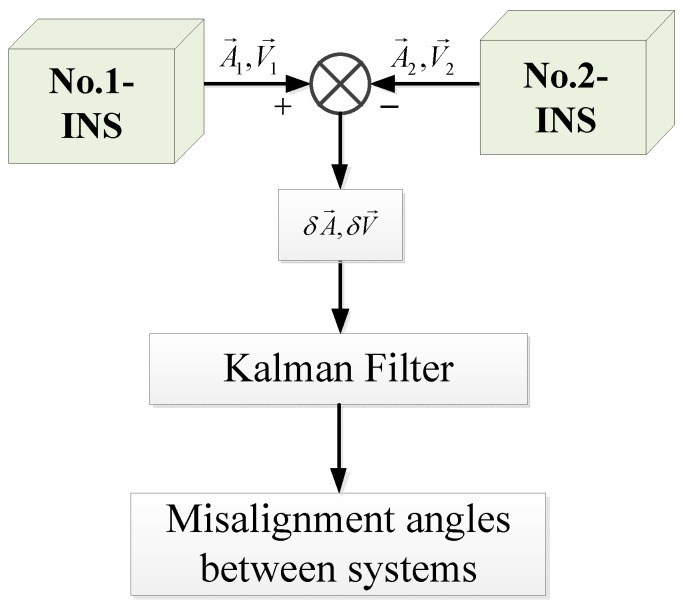
The misalignment angles calibration scheme.

In this paper, the misalignment angles between two inertial navigation systems are known as small angles. This method calibrates misalignment angles between systems by the navigation information of two inertial navigation systems. A mathematical model was built according to the relationship of misalignment angles and the speed and attitude difference of two systems. then a Kalman filter was designed to estimate the misalignment angles between systems. The scheme is shown in Figure 3.

### 3.2. Inertial Device Biases Model

Inertial devices biases are the main source of inertial navigation error, and it seriously affects the navigation accuracy of the inertial navigation system [19]. In order to make simulation experiments closer to reality, the error model of the inertial device should be established before the accurate calibration of the misalignment angles between systems. In general, the inertial device biases consists of Gaussian white noise, the random constant bias, and the first-order Markov process [20]. The white Gaussian noise describes the fast changing characteristics of the deviation. The Markov process describes the slow-varying characteristics of the deviation, which is more significant for INS long-term endurance navigation. The inertial device error model can be expressed as:

(6) $$\{\begin{array}{l} \varepsilon_{g}=\varepsilon_{b}+\varepsilon_{r}+w_{g}\;\\ {\dot{\varepsilon }}_{r}=-\frac{1}{\tau_{G}}\varepsilon_{r}+w_{gr}\\ {\dot{\varepsilon }}_{b}=0 \end{array}$$

(7) $$\{\begin{array}{l} \nabla_{a}=\nabla_{b}+\nabla_{r}+w_{a}\;\\ {\dot{\nabla }}_{r}=-\frac{1}{\tau_{A}}\nabla_{r}+w_{ar}\\ {\dot{\nabla }}_{b}=0\; \end{array}$$

where $\varepsilon_{b}$ and $\nabla_{b}$ denote random constant components of gyro drifts and accelerometer biases respectively. $\varepsilon_{r}$ and $\nabla_{r}$ are the first-order Markov processes, in which $\tau_{G}$ and $\tau_{A}$ denote the related time, $w_{gr}$ and $w_{ar}$ are white noises of slow-varying biases, respectively. $w_{g}$ and $w_{a}$ are white Gaussian noises of white noises gyro drifts and accelerometer biases. In this paper, the calibration process is fast, so the influence of the first-order Markov process can be omitted. The simplified error model is:

(8) $$\{\begin{array}{l} \varepsilon_{g}=\varepsilon_{b}+w_{g}\;\\ {\dot{\varepsilon }}_{b}=0 \end{array}$$

(9) $$\{\begin{array}{l} \nabla_{a}=\nabla_{b}+w_{a}\;\\ {\dot{\nabla }}_{b}=0 \end{array}$$

### 3.3. System Model Established

**Figure 4:**
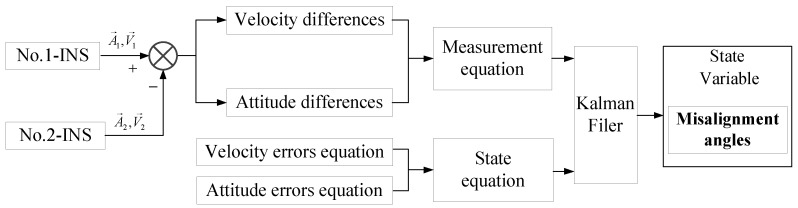
Design of Kalman filter.

The Kalman filter designed to estimate the misalignment angles between systems is shown in Figure 4.

Selecting the platform angles, velocity error, position error, gyroscope drift, accelerometer bias, and misalignment angles between two systems as the state variable. The velocity error and attitude error equations are chosen as state equation, which is shown as below:

First of all, the velocity error equations are presented, as follows:

(10) $$\begin{array}{l} \delta {\dot{V}}_{E}=-\delta \phi_{N}f_{U}+\delta \phi_{U}f_{N}+\frac{V_{N}\tan L\;}{R}\delta V_{E}+(2\omega_{ie}\sin L+\frac{V_{E}}{R}\tan L)\delta V_{N}+(2\omega_{ie}\cos LV_{N}+\frac{V_{E}V_{N}}{R}\sec^{2}L)\delta L+\Delta \nabla_{E}\\ \delta {\dot{V}}_{N}=\delta \phi_{E}f_{U}-\delta \phi_{U}f_{E}-2(\omega_{ie}\sin L+\frac{V_{E}}{R}\tan L)\delta V_{E}-(2\omega_{ie}\cos LV_{E}+\frac{V_{E}^{2}}{R}\sec^{2}L)\delta L+\Delta \nabla_{N} \end{array}$$

In Equation (10), $\delta V_{E},\delta V_{N},\delta V_{U}$ denote velocity errors of No.1-INS and the No.2-INS, $\delta \phi_{E},\delta \phi_{N},\delta \phi_{U}$ are platform angles, δL,δλ are position error, $f_{E},f_{N},f_{U}$ are the specific forces in n-frame, and $\Delta \nabla_{E},\Delta \nabla_{N},\Delta \nabla_{U}$ are equivalent accelerometer biases in n-frame.

Secondly, attitude error equations are shown, as follows:

(11) $$\begin{array}{l} \delta {\dot{\phi }}_{E}=\delta \phi_{N}(\omega_{ie\;}\sin L+\frac{V_{E}}{R}\tan L)-\delta \phi_{U}(\omega_{ie}\cos L+\frac{V_{E}}{R})-\frac{\delta V_{N}}{R}+\Delta \varepsilon_{E}\\ \delta {\dot{\phi }}_{N}=-\delta \phi_{E}(\omega_{ie}\sin L+\frac{V_{E}}{R}\tan L)-\delta \phi_{U}\frac{V_{N}}{R}-\omega_{ie}\sin L\delta L+\frac{\delta V_{E}}{R}+\Delta \varepsilon_{N}\\ \delta {\dot{\phi }}_{U}=\delta \phi_{E}(\omega_{ie}\cos L+\frac{V_{E}}{R})+\delta \phi_{N}\frac{V_{N}}{R}+(\omega_{ie}\cos L+\frac{V_{E}}{R}\sec^{2}L)\delta L+\frac{\delta V_{E}}{R}\tan L+\Delta \varepsilon_{U} \end{array}$$

where $\Delta \varepsilon_{E},\Delta \varepsilon_{N},\Delta \varepsilon_{U}$ are equivalent gyro drifts in n-frame.

State variables were:

(12) $$X={\left[\begin{array}{llllllllllllllll} \delta V_{E}\; & \delta V_{N} & \delta \phi_{E} & \delta \phi_{N} & \delta \phi_{U} & \delta \lambda & \delta L & \Delta \varepsilon_{x} & \Delta \varepsilon_{y} & \Delta \varepsilon_{z} & \Delta \nabla_{x} & \Delta \nabla_{y} & \Delta \nabla_{z} & \Delta \theta & \Delta \gamma & \Delta \psi \end{array}\right]}^{T}$$

Because the matrix $C_{b2}^{n^{\prime }}$, matrix $C_{n}^{n\prime }$, matrix $C_{b1}^{n}$, and the matrix $C_{b1}^{b2}$ have following relationship:

(13) $$C_{b2\;}^{n^{\prime }}=C_{n}^{n\prime }C_{b1}^{n}C_{b1}^{b2}=(I-E)C_{b1}^{n}(I-\Lambda )$$

In the above equation:

(14) $$\Lambda =\left[\begin{array}{ccc} 0 & -\Delta \psi \; & \Delta \lambda \\ \Delta \psi & 0 & -\Delta \theta \\ -\Delta \lambda & \Delta \theta & 0 \end{array}\right]$$

(15) $$C_{b2\;}^{n^{\prime }}={\left(R_{2}(\gamma_{2})R_{1}(\theta_{2})R_{3}(\psi_{2})\right)}^{T}=\left[\begin{array}{ccc} S_{11}^{\prime } & S_{12}^{\prime } & S_{13}^{\prime }\\ S_{21}^{\prime } & S_{22}^{\prime } & S_{23}^{\prime }\\ S_{31}^{\prime } & S_{32}^{\prime } & S_{33}^{\prime } \end{array}\right]$$

(16) $$C_{b1\;}^{n}={\left(R_{2}(\gamma_{1})R_{1}(\theta_{1})R_{3}(\psi_{1})\right)}^{T}=\left[\begin{array}{ccc} S_{11} & S_{12} & S_{13}\\ S_{21} & S_{22} & S_{23}\\ S_{31} & S_{32} & S_{33} \end{array}\right]$$

Substituting Equations (14)–(16) into Equation (13), the relationship between attitude differences of two INSs and platform angles, misalignment angles between two systems can be calculated:

(17) $$\delta \theta =\frac{S_{12\;}}{\sqrt{1-S_{32}^{2}}}\delta \phi_{N}-\frac{S_{22}}{\sqrt{1-S_{32}^{2}}}\delta \phi_{E}-\frac{S_{31}}{\sqrt{1-S_{32}^{2}}}\Delta \psi +\frac{S_{33}}{\sqrt{1-S_{32}^{2}}}\Delta \theta$$

(18) $$\delta \gamma =\frac{S_{21\;}S_{33}-S_{23}S_{31}}{S_{31}^{2}+S_{33}^{2}}\delta \phi_{E}+\frac{S_{13}S_{31}-S_{11}S_{33}}{S_{31}^{2}+S_{33}^{2}}\delta \phi_{N}+\frac{S_{32}S_{31}}{S_{31}^{2}+S_{33}^{2}}\Delta \theta +\Delta \gamma -\frac{S_{32}S_{33}}{S_{31}^{2}+S_{33}^{2}}\Delta \psi$$

(19) $$\delta \psi =-\delta \phi_{U}+\frac{S_{32\;}S_{22}}{S_{12}^{2}+S_{22}^{2}}\delta \phi_{N}+\frac{S_{12}S_{32}}{S_{12}^{2}+S_{22}^{2}}\delta \phi_{E}-\frac{S_{13}S_{22}-S_{23}S_{12}}{S_{12}^{2}+S_{22}^{2}}\Delta \theta +\frac{S_{11}S_{22}-S_{12}^{2}}{S_{12}^{2}+S_{22}^{2}}\Delta \psi$$

The filter selects the velocity differences and attitude differences of No.1-INS and No.2-INS as the measurement to achieve the best estimate of the misalignment angles between the two systems. The measurement equation can be obtained from Equations (17)–(19):

(20) $$\begin{array}{ll} Z & =HX={\left[V_{A\_INS1\;}-V_{A\_INS2};A_{INS1}-A_{INS2}\right]}^{T}\\ & ={\left[\begin{array}{lllll} \delta V_{E} & \delta V_{N} & \Delta \theta & \Delta \gamma & \Delta \psi \end{array}\right]}^{T} \end{array}$$

(21) $$H=\left[\begin{array}{llll} A & 0_{2\times 3\;} & 0_{2\times 8} & 0_{2\times 3}\\ 0_{3\times 2} & B & 0_{3\times 8} & C \end{array}\right]$$

where:

(22) $$A=\left[\begin{array}{cc} 1 & 0\\ 0 & 1 \end{array}\;\right]$$

(23) $$B=\left[\begin{array}{ccc} -\frac{S_{22\;}}{\sqrt{1-S_{32}^{2}}} & \frac{S_{12}}{\sqrt{1-S_{32}^{2}}} & 0\\ \frac{S_{21}S_{33}-S_{23}S_{31}}{S_{31}^{2}+S_{33}^{2}} & \frac{S_{13}S_{31}-S_{11}S_{33}}{S_{31}^{2}+S_{33}^{2}} & 0\\ \frac{S_{12}S_{32}}{S_{12}^{2}+S_{22}^{2}} & \frac{S_{32}S_{22}}{S_{12}^{2}+S_{22}^{2}} & -1 \end{array}\right]$$

(24) $$C=\left[\begin{array}{ccc} \frac{S_{33\;}}{\sqrt{1-S_{32}^{2}}} & 0 & -\frac{S_{31}}{\sqrt{1-S_{32}^{2}}}\\ \frac{S_{32}S_{31}}{S_{31}^{2}+S_{33}^{2}} & 1 & -\frac{S_{32}S_{33}}{S_{31}^{2}+S_{33}^{2}}\\ -\frac{S_{13}S_{22}-S_{23}S_{12}}{S_{12}^{2}+S_{22}^{2}} & 0 & \frac{S_{11}S_{22}-S_{12}^{2}}{S_{12}^{2}+S_{22}^{2}} \end{array}\right]$$

## 4. Observability Analysis

### 4.1. PWCS (Piece-Wise Constant System) Observability Analysis

In the previous section, a Kalman filter was established to estimate the misalignment angles between two systems from a theoretical perspective. In order to further prove the effectiveness of the filter, an observability analysis of the state variables is required. According to observability theory, it is known that the time-varying system observability matrix that was used in this paper is difficult to analyze [21], but considering that the INS update interval is short enough, in this case, the time-varying system can be regarded as a linear fixed-length system [22]. Therefore, this paper uses the PWCS method to verify the observability of the above scheme. According to the PWCS method, the observable matrix corresponding to each time interval can be expressed, as follows:

(25) $${\tilde{Q}}_{i}={\left[\begin{array}{ccccc} {(H_{i})\;}^{T} & {\left(H_{i}F_{i}\right)}^{T} & {\left(H_{i}F_{i}^{2}\right)}^{T} & \cdots & {\left(H_{i}F_{i}^{n-1}\right)}^{T} \end{array}\right]}^{T}$$

where i is the time index of each interval, $H_{i}$ is the measurement matrix, and $F_{i}$ is the state transition matrix. The $H_{i}$ and $F_{i}$ matrix can be obtained according to measurement equations and state equations of the Kalman filter model of Section 3.

The total observability matrix (TOM) of the PWCS is defined, as follows:

(26) $$\tilde{Q}(r)=\left[\begin{array}{c} {\tilde{Q}}_{1}\;\\ {\tilde{Q}}_{2}e^{F_{1}\Delta_{1}}\\ \vdots \\ {\tilde{Q}}_{r}e^{F_{r-1}\Delta_{r-1}}\cdots e^{F_{1}\Delta_{1}} \end{array}\right]$$

where r is the number of time intervals. In order to analyze the observability more convenient, generally, the TOM could be replaced by Stripped Observability Matrix (SOM), which is shown as:

(27) $${\tilde{Q}}_{s}(r)={\left[\begin{array}{cccc} {\tilde{Q}}_{1}\; & {\tilde{Q}}_{2} & \cdots & {\tilde{Q}}_{r} \end{array}\right]}^{T}$$

When $rank({\tilde{Q}}_{s})=n$, the defined PWCS is fully observable, but if $rank({\tilde{Q}}_{s})<n$ the system is not fully observable. In this paper, state variables are 13 dimensions and the rank of ${\tilde{Q}}_{s}$ is also 13, thus the system is full observable.

### 4.2. SVD (Singular Value Decomposition) Observable Degree Analysis

The PWCS method cannot realize quantitative analysis in specific. In order to indicate the observable degree of state variables the SVD method is needed [23]. In the singular value decomposition method the matrix ${\tilde{Q}}_{s}$ could be decomposed, as follows:

(28) $${\tilde{Q}}_{s}=U\Sigma V^{T}\;$$

where $U=\left[\begin{array}{cccc} u_{1} & u_{2} & \cdots & u_{m} \end{array}\right]$, $V=\left[\begin{array}{cccc} v_{1} & v_{2} & \cdots & v_{m} \end{array}\right]$ are both orthogonal matrixes. Also, $\Sigma =\left[\begin{array}{c} S\\ O_{(m-r)\times r} \end{array}\right]$ is a matrix of m×r orders. Besides, $S=diag\left\{\begin{array}{cccc} \sigma_{1} & \sigma_{2} & \cdots & \sigma_{r} \end{array}\right\}$ is a diagonal matrix, and $\sigma_{1}\geq \sigma_{2}\geq \cdots \geq \sigma_{r}\geq 0$ are defined as singular values of ${\tilde{Q}}_{s}$. According to SVD decomposition, the measurable variables can be expressed as:

(29) $$Z=\sum_{j=1\;}^{r}\sigma_{j}(v_{j}^{T}X_{0})u_{j}$$

If $\sigma_{r}>0$, the initial state of variable $X_{0}$ could be evaluated based on measurements, which can be shown, as follow:

(30) $$X_{0}={(U\Sigma V^{T})\;}^{-1}Z=\sum_{j=1}^{r}(\frac{u_{j}^{T}z}{\sigma_{j}})v_{j}$$

From Equation (30), it can be seen that the required initial value is smaller when the singular value of the state variable is bigger, which means that the observable degree of the state variable is higher [24]. On the contrary, if the singular values are too small the state variable will difficult to be observed.

**Figure 5:**
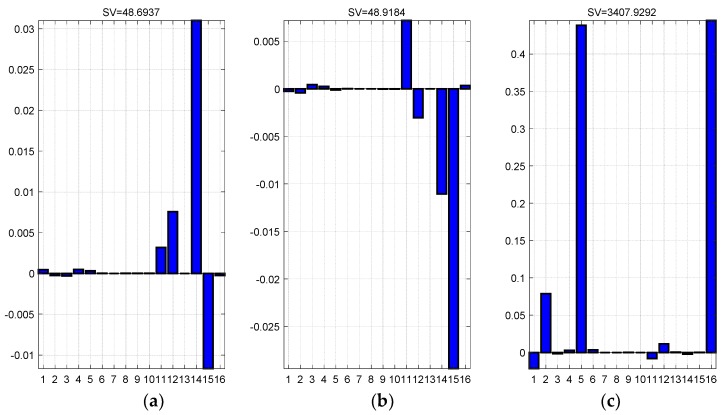
Observability analysis results of state variables. (**a**) Description of the Δθ observability; (**b**) Description of the Δγ observability; and, (**c**) Description of the Δψ observability.

Based on the PWCS method and the SVD method, the observability of state variables is analyzed qualitatively and quantitatively. Analysis results are shown in Figure 5.

Figure 5a–c are the observability analysis results of Δθ, Δγ and Δψ misalignment angles. It could be seen that the singular values of above three angles are basically at a relative large level, which indicates the high degrees of observable characteristics. This means that the Kalman filter can estimated misalignment angles between systems fast and accuracy.

## 5. Simulation Results and Analysis

**Figure 6:**
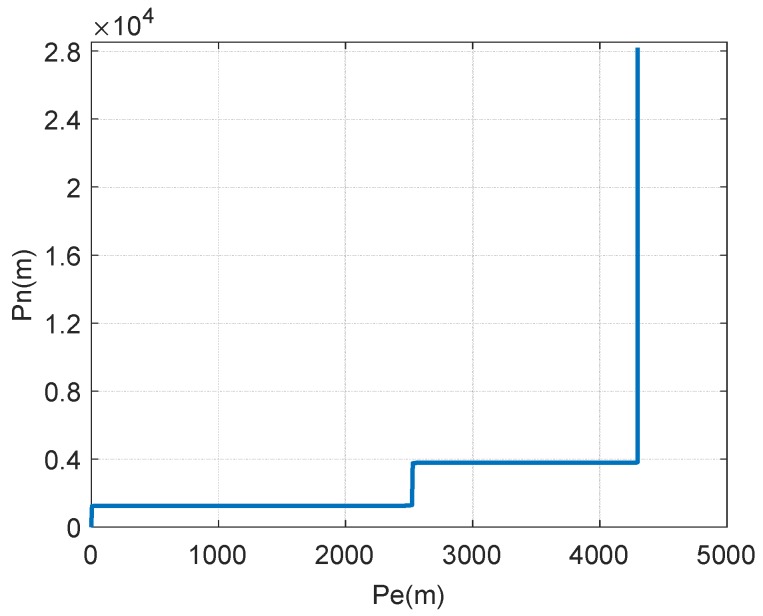
Vehicle trajectory.

In order to prove the effectiveness of this method, a dynamic simulation experiment was carried out. The simulation considers the common maneuvering, including uniform motion, accelerate motion, decelerate motion heading angle change, and pitching change during the driving of the vehicle. Since the rolling change is little during the maneuver of the vehicle, it could be ignored in this paper. The specific maneuvering of the vehicle is shown as Figure 6.

**Figure 7:**
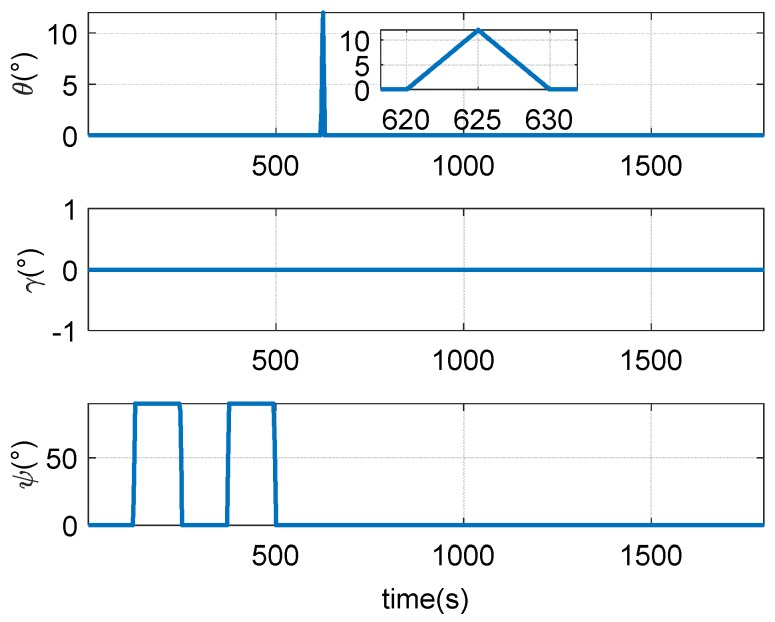
The attitude change of the vehicle.

The attitude change during the driving is shown in the Figure 7.

**Figure 8:**
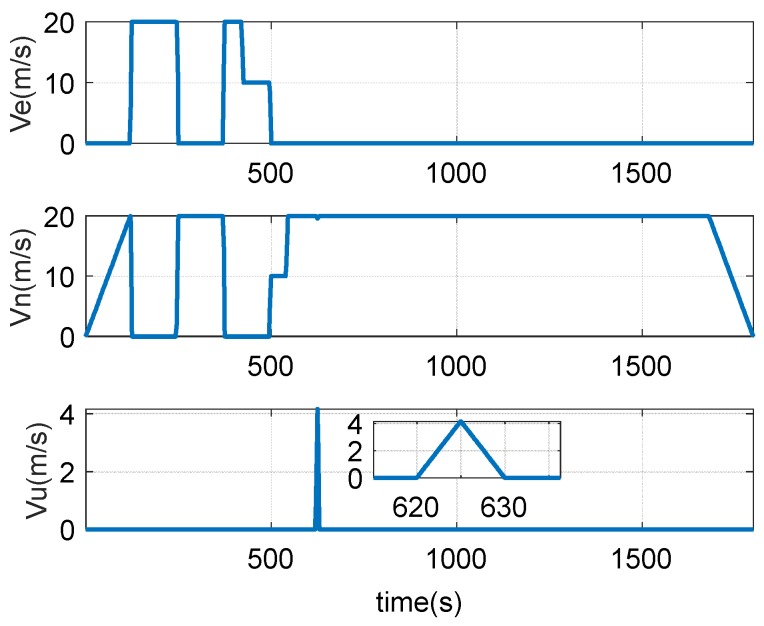
Vehicle speed change of the vehicle.

Figure 8 shows the speed change during driving, including east speed, north speed, and up speed.

Due to the measurement error of the inertial device in practical applications, according to the actual situation, various errors of the inertial device should be added to the simulation data generated by the trajectory generator, including gyro drift, accelerometer bias, random white noise, etc. The detailed simulation settings of inertial device biases and the misalignment angles settings of the two INSs are provided in Table 1.

Based on the dynamic trajectory simulated abovementioned several dynamic simulation experiments are performed and the performance of the proposed method is further tested. One of the calibration results of the misalignment angles between the systems are shown in below.

**Figure 9:**
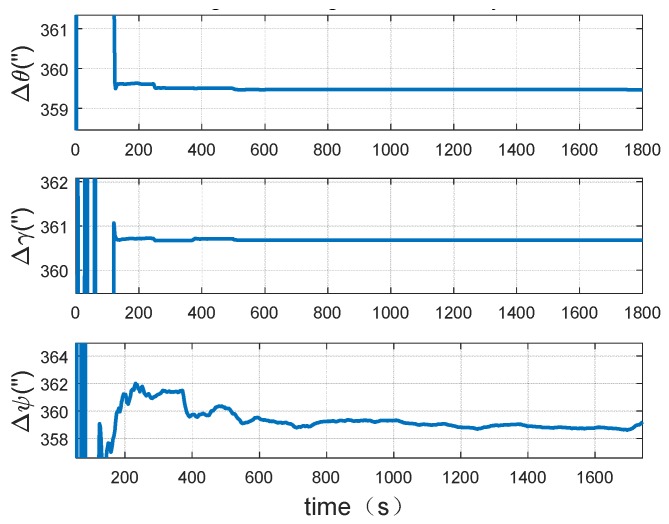
Dynamic simulation result of misalignment angles between systems.

**Figure 10:**
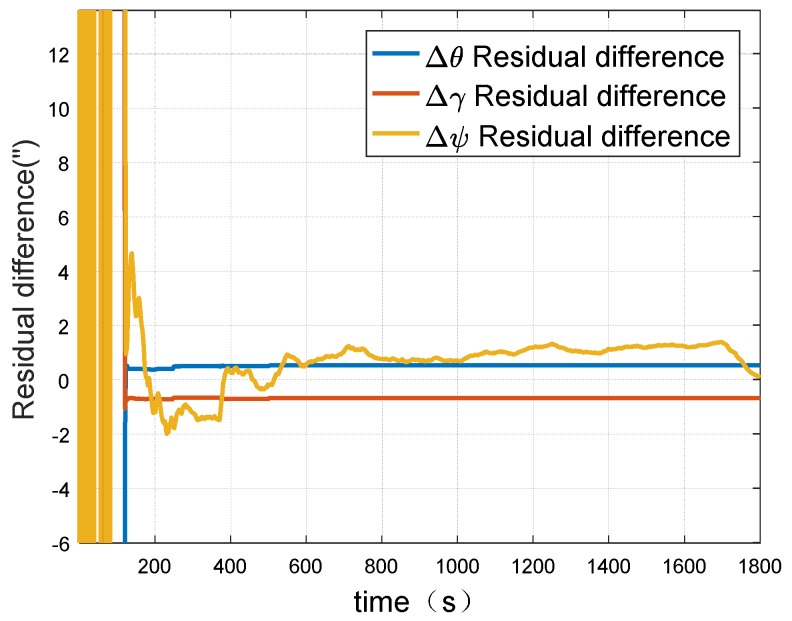
Residual difference between estimate value and setting value of misalignment angles between two inertial navigation systems (INSs).

It could be seen from Figure 9 and Figure 10 that in the simulation experiment the misalignment angles between systems could be well calibrated. The accuracy of misalignment angle in azimuth direction is 2″. Moreover, the misalignment angles of level direction have higher accuracy than the angle in azimuth direction. In addition, it can be found that the misalignment angles can converge to the exact value within 200 s. The convergence speed is fast.

## 6. Experimental Results of Dynamic Calibration

In order to further demonstrate the effectiveness of the proposed method in actual application, an experiment with the dynamic calibration method is performed with INSs that were manufactured by our lab.

During the calibration process, two INSs are mounted on a transition board where the b-frame are not completely coincident. The transition board is placed on a small trailer in the laboratory. The experimenter dragged the small trailer to simulate the maneuver of the launch vehicle. Navigation data of two INSs is collected by a laptop at the frequency of 200 Hz. 

**Figure 11:**
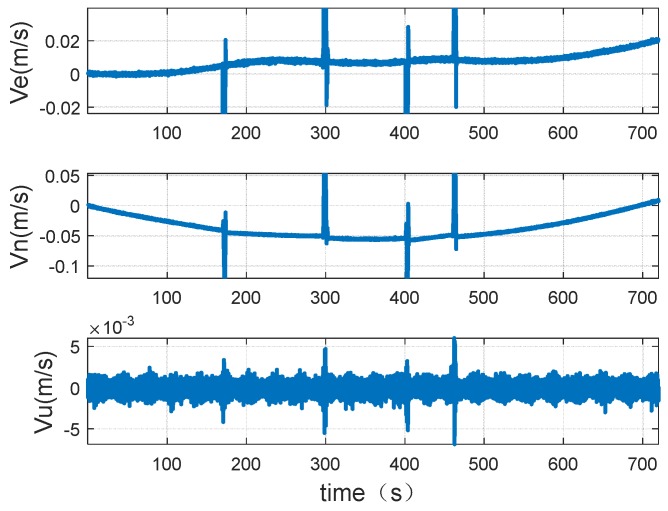
Speed of the trailer.

**Figure 12:**
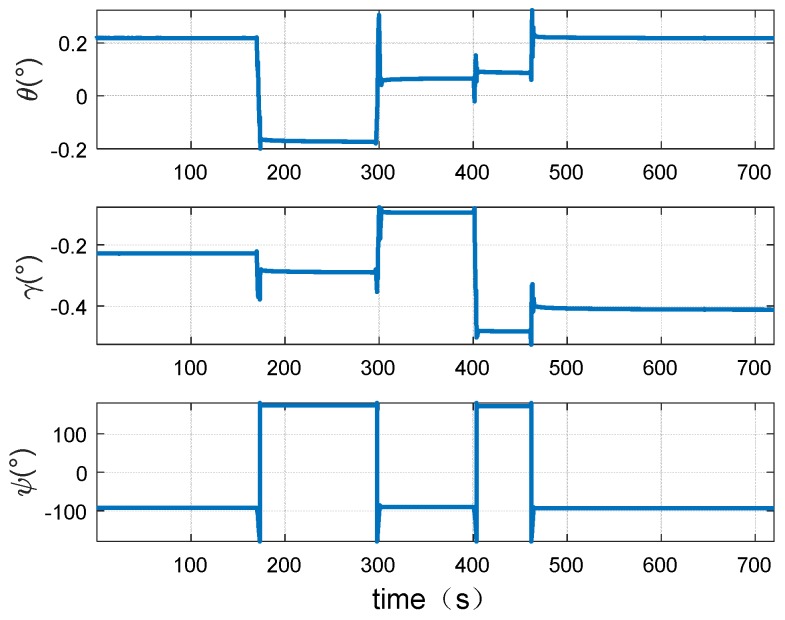
The attitude change of the trailer.

The attitude and speed changes during the experiment are shown in Figure 11 and Figure 12.

**Figure 13:**
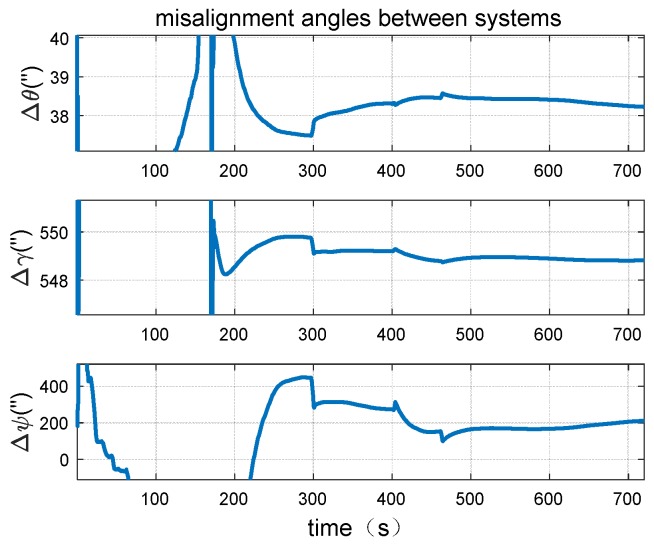
Dynamic calibration results of misalignment angles between systems.

**Figure 14:**
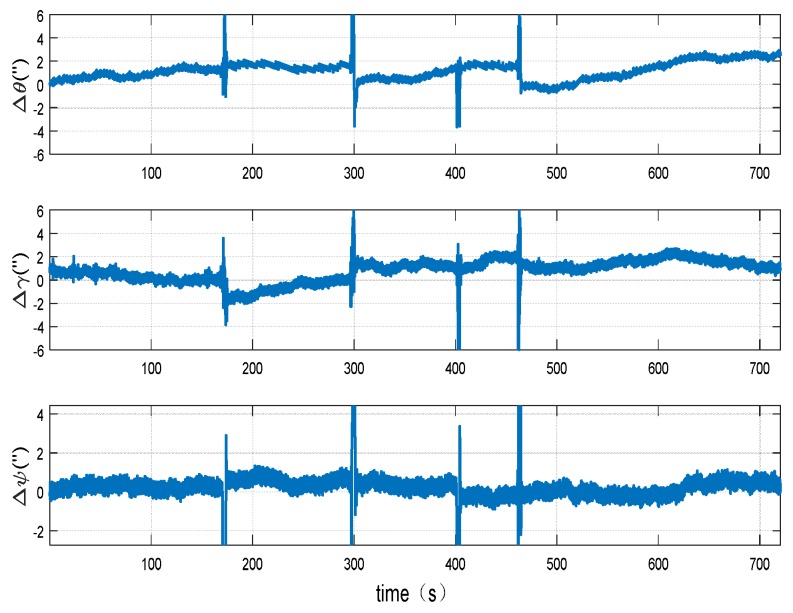
Attitude difference of two INSs after compensated misalignment angles.

The calibration results of the misalignment angles between the systems are shown in Figure 13 and Figure 14.

It can be seen from Figure 13 that, through the proposed dynamic calibration method, the misalignment angles between systems on horizontal direction can converge within the period of 300 s and the misalignment angles between systems on vertical direction can converge within the period of 500 s, which is in accordance with the simulation result. According to the two INSs attitude difference after comparison figure (Figure 14), it can be clearly seen that the attitude differences of two INSs significantly reduced after misalignment angles compensation. The peak-to-peak value of the pitch angle difference of two INSs after compensation is within 3″, the peak-to-peak value of the rolling angle difference is within 4″and the peak-to-peak value of the heading angle difference is within 2″. As in this experiment, the maneuver angle of the vehicle is about 90°, the maximum attitude difference after misalignment angles compensation is 4″. Divide the maximum attitude difference by the maneuver angle, the misalignment angles can be calculated. Thus, it can be concluded that the calibration accuracy of misalignment angles could be better than 1″ by using the proposed method.

## 7. Conclusions

In this paper, a dynamic calibration method for misalignment angles between two sets of inertial navigation systems is proposed. In order to verify the effectiveness of this method, a simulation and INSs experiments were conducted. The simulation results show that all of the misalignment angles can tend to the exact value within 200s and the precision of misalignment angles can be limited within 1″ while using the proposed method. The INSs experiment shows that the misalignment angles between two sets of systems can converge within 300s by the dynamic calibration method that was proposed in this paper and the installation misalignment angles between systems can be limited to 1″. The proposed method can calibrate the misalignment angles between the two sets of systems with the maneuver of the vehicle. Therefore, it has high significance on the occasions where information fusion is needed of multiple inertial navigation systems.

## Figures and Tables

**Table 1:** Settings of inertial device biases and misalignment angles.

Parameters		Settings
Gyro drifts	Gyro constant drifts	$0.01^{\circ }/h$
	Random walk	$0.001^{\circ }/\sqrt{h}$
Accelerometer biases	Accelerometer constant biases	50μg
	Random walk	$0.2\mu g\cdot \sqrt{h}$
Misalignment angles	Δθ	$0.1^{\circ }$
	Δγ	$0.1^{\circ }$
	Δψ	$0.1^{\circ }$
